# Willingness to Pay for Pharmacist-Led Weight Management Services in Community Pharmacies: A Cross-Sectional Study in an Academic Medical Center in Saudi Arabia

**DOI:** 10.3390/healthcare14131953

**Published:** 2026-07-02

**Authors:** Saja H. Almazrou, Danah Alwakail, Felwah Almanea, Shiekha S. AlAujan

**Affiliations:** Department of Clinical Pharmacy, College of Pharmacy, King Saud University, Riyadh 11362, Saudi Arabia; 442200746@student.ksu.edu.sa (D.A.); 442201264@student.ksu.edu.sa (F.A.); salaujan@ksu.edu.sa (S.S.A.)

**Keywords:** willingness to pay, weight management, community pharmacy

## Abstract

**Background:** Obesity is a growing public health crisis in Saudi Arabia. Community pharmacists are well-positioned to deliver accessible weight management services, but data on public willingness to pay (WTP) for these interventions remains limited. **Aim:** To assess the willingness of the Saudi Arabian public to pay for community pharmacist-led weight management services and identify factors associated with WTP. **Methods:** A cross-sectional, face-to-face survey was conducted among adults in a medical city in Riyadh between October 2025 and January 2026. WTP was elicited using the Payment Card method based on a hypothetical pharmacist-led service scenario. Payment values were validated by an expert panel. Univariable and multivariable regression models were used to identify independent predictors of WTP and payment amounts. **Results:** Of 746 participants, 66% expressed willingness to pay for the service. The most frequently selected maximum payment was 50 SAR per session. Multivariable logistic regression revealed that WTP was significantly associated with younger age, higher monthly income, perceived usefulness of pharmacy services (aOR: 3.10; 95% CI: 1.83–5.26), frequent pharmacy visits, and prior or desired access to a dietitian. Clinical burden, including BMI and chronic conditions, did not significantly influence WTP. Among those willing to pay, male gender was independently associated with a lower stated payment amount compared to females (β = −3.8 SAR; *p* = 0.006). **Conclusions:** Among adults attending a large academic medical city in Riyadh, there is substantial willingness to pay for pharmacist-led weight management services, with perceived value and healthcare engagement as the primary drivers. These preliminary findings warrant replication in broader, nationally representative samples.

## 1. Introduction

Obesity is a complex, chronic disease marked by excessive fat accumulation that harms health and shortens life expectancy. Defined in adults as a BMI ≥ 30 kg/m^2^, it is now seen as a multifactorial public health issue involving genetics, metabolism, sociocultural factors, and lifestyle changes [1]. As of 2022, over 1 billion people, 650 million adults, 340 million adolescents, and 39 million children were living with obesity, nearly triple the 1975 figures. This widespread obesity is strongly associated with non-communicable diseases: 67.6% of individuals with obesity have hypertension, 60.7% have type 2 diabetes, and 51.3% have hypercholesterolemia [2]. Losses from reduced productivity and early mortality were reported [1]. The economic impact is substantial, with annual healthcare costs estimated at SAR 24 billion [2].

Despite increased awareness, current prevention and management strategies remain insufficient, often fragmented, and poorly integrated into primary healthcare. Sociocultural factors, including dietary patterns, sedentary lifestyles, and body image perceptions, further complicate the challenge, highlighting the urgent need for culturally tailored, community-based interventions [3,4].

Pharmacists located in community-based pharmacies, as highly accessible and trusted healthcare providers, are well-positioned to support obesity management. Their evolving role increasingly extends beyond dispensing medication to include direct patient care services such as chronic disease management, lifestyle counseling, and preventive health education [5,6]. A systematic review on community pharmacy-based interventions have shown significant effects on weight reduction and associated cardiovascular risk factors, with studies showing average weight losses of 5.3 kg in females and 6.2 kg in males, sustained at one-year follow-up. Such interventions offer the advantage of frequent patient contact, convenience, and cost-effectiveness, making them a valuable strategy to augment traditional healthcare models and address obesity at a population level. However, implementing these interventions successfully requires more than strong clinical evidence; it also depends on public acceptance, perceived value, and willingness to participate. Willingness to pay (WTP) studies offer important insights into how individuals value health services, supporting economic evaluations and guiding service design [7,8].

Within the community pharmacy setting, only two studies investigate the WTP for weight management services [9,10]. A cross-sectional study in Jordan evaluated the public WTP for a hypothetical pharmacist-led weight management program using a payment card method administered via face-to-face questionnaires. The study found that 85.2% of respondents (n = 966) indicated WTP, with a mean of $17.20 per visit [10].

Another study in Australia employed an online cross-sectional survey (n = 403) and found that only 13.9% of participants had previously sought pharmacists’ advice for weight management; however, those with prior engagement were significantly more supportive and more willing to pay for future services. About half of respondents were unwilling to pay, but among those willing, amounts ranged from AU$10 to AU$50 per session, with a median of AU$10 [9]. While regional evidence such as the Jordanian study indicates high acceptability, Saudi Arabia presents a unique economic and healthcare landscape undergoing rapid transformation. Therefore, local data is essential to establish the novelty and applicability of these services in the Saudi context.

In Saudi Arabia, obesity has become a rapidly growing public health crisis. National data show adult obesity prevalence between 20% and 39%, with adolescent rates reaching up to 19.4% [2]. While WTP has been explored for other pharmacist-led services in Saudi Arabia, including medication counseling [11] and medication therapy management [12] no study has assessed willingness to pay specifically for pharmacist-led weight management services in the Kingdom. This gap limits evidence-based resource allocation and the design of service models aligned with patient preferences and cultural norms within the Saudi healthcare system. Understanding this can provide preliminary insights for policy development, resource allocation, and designing service models that align with patient preferences and cultural norms. Therefore, this study aims to assess the willingness of the Saudi Arabian public to pay for pharmacist-led weight management services offered in community pharmacies. WTP methodology was selected because it provides a validated approach for quantifying the monetary value individuals assign to health services prior to their implementation, thereby informing reimbursement decisions, service pricing, and policy planning within the Saudi healthcare system.

## 2. Methods

### 2.1. Study Design

This cross-sectional study used a quantitative, face-to-face survey to assess consumers’ WTP for community pharmacist-led weight management services in Saudi Arabia. Data were collected from adult consumers (≥18 years) in waiting areas in King Saud University Medical City (KSUMC) in Riyadh using convenience sampling. Trained pharmacy students and research assistants recruited participants during clinic hours. Sample size was calculated using the Raosoft^®^ calculator (95% confidence level, 5% margin of error) yielding a sample of 377 [13]. To increase statistical power and account for potential non-responses, we aimed to recruit a larger sample.

Eligible participants were Arabic- or English-speaking Riyadh residents who provided informed consent. Individuals under 18 or with cognitive impairments were excluded. The data was collected between October 2025 and January 2026. This cross-sectional study was conducted in adherence with the Strengthening the Reporting of Observational Studies in Epidemiology (STROBE) guidelines [14].

### 2.2. Study Instrument

A structured questionnaire was developed using health economics principles, expert consensus, and the published literature [9,10,15]. The instrument comprised five sections: demographics and socioeconomic characteristics; access to obesity treatments; experiences and perceptions of community pharmacy services; expectations and service preferences; and a WTP assessment. WTP was elicited using the payment card method within a hypothetical scenario describing a pharmacist-led weight management service, including demonstrated clinical benefits, consultation details, lifestyle counseling, and body composition analysis, excluding medication costs. The full survey is provided in Supplementary File S1.

The questionnaire was originally developed in English and translated into Arabic to ensure accessibility for Arabic-speaking participants. The Arabic version was reviewed by bilingual experts in pharmacy practice to verify linguistic accuracy and conceptual clarity prior to use. Data were collected through face-to-face, interviewer-administered questionnaires, whereby trained pharmacy students and research assistants read the items to participants and recorded their responses. Face and content validity for the entire questionnaire were evaluated by a panel of community pharmacists and academics in pharmacy practice, who assessed each item for clarity, relevance, and appropriateness to the target population. Feedback was incorporated iteratively until consensus was reached on the final instrument. The questionnaire was adapted from the previously published literature on WTP for pharmacy services [9,10,15].

Payment card values were determined through an expert panel meeting to ensure the proposed range and increments were appropriate for the Saudi context and minimized anchoring or ceiling/floor biases. This panel included five professionals with insights into the Saudi healthcare system, such as experts from the National Unified Procurement Company (NUPCO), the Cooperative Health Insurance (CHI) council, insurance companies and academics.

### 2.3. Ethical Approval

Ethical approval was obtained from the King Saud University Medical City IRB (project number E-25-10075). Participation was voluntary, anonymous, and uncompensated, with a collective charitable donation made on participants’ behalf.

### 2.4. Statistical Analysis

All analyses were conducted using R version 2026.01.0+392. Participant characteristics were summarized using descriptive statistics. Categorical variables are presented as number (percentage) and continuous variables as mean (standard deviation).

Factors associated with WTP were examined using logistic regression. Univariable logistic regression models were first fitted. Variables with *p* < 0.20 in univariable analysis, together with variables considered clinically relevant, were considered for inclusion in the multivariable model; this liberal screening threshold was used to avoid the premature exclusion of potentially important predictors that a stricter 0.05 criterion may miss. All selected variables were then simultaneously entered into the multivariable logistic regression model using the Enter (forced entry) method [16,17].

A multivariable logistic regression model was then fitted to estimate adjusted odds ratios (aORs) and 95% CIs for factors associated with WTP. As a secondary analysis, factors associated with the maximum stated amount willing to be paid per session were examined among participants reporting a positive payment amount using univariable and multivariable linear regression. Participants with missing values or a reported amount of 0 SAR were excluded. Results are presented as regression coefficients (β) with 95% confidence intervals (CIs). Alpha level < 0.05 was considered statistically significant.

## 3. Results

Of the 800 individuals approached for the study, 746 agreed to participate, yielding a response rate of 95.5%. Following data cleaning, 746 participants who completed the survey were included in the final analysis. The study cohort was predominantly females (64%) and relatively young, with nearly half (45%) aged under 25 years. The sample was highly educated, as 74% held a university degree, and the vast majority (95%) resided in urban areas. In terms of weight status, approximately 45% of participants were classified as overweight or obese (Table 1). A quarter of the respondents (25%) reported having at least one chronic health condition, with diabetes, hypertension, and asthma being the most prevalent (Supplementary File S2).

Community pharmacies were a regular point of healthcare access, with nearly half of the participants visiting a pharmacy at least monthly. While only 15% had previously asked a pharmacist for advice regarding weight, diet, or lifestyle, a substantial portion of the cohort (40%) perceived pharmacy-based weight management services as potentially useful. When considering the format of such services, participants demonstrated a strong preference for traditional, in-person, one-on-one consultations (49%) over virtual or telehealth alternatives (32%). Expectations for these consultations centered on practical and measurable support, with lifestyle recommendations, body weight and BMI monitoring, and nutritional advice being the most highly anticipated components (Supplementary File S3).

The use of medical interventions for weight loss was limited within the cohort. Only 4.6% of participants reported current use of prescription weight-management medications, primarily GLP-1 or dual GIP/GLP-1 receptor agonists. Similarly, a small minority (13%) had previously undergone formal weight-loss procedures, such as bariatric surgery or medically supervised diets (Supplementary File S4).

When asked about ongoing weight management support, more than half of the participants (51%) indicated they were not receiving any structured assistance. Among those who did utilize support systems, digital tools (online programs or apps) were the most common, followed by consultations with physicians or registered dietitians (Table 2).

**Figure 1:**
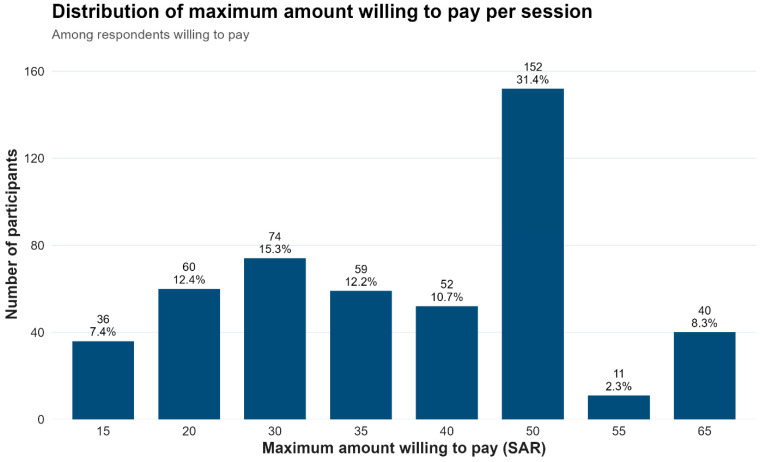
Maximum amount of WTP per session.

Overall, 66% of the cohort indicated they would be willing to pay for a pharmacy-based weight management program. Among the remaining 34% who declined, the primary reasons were that they were already managing their weight independently (39%), lack of interest (21%), and financial or insurance concerns (21%). For those amenable to the service, expected per-session costs varied, with 50 SAR being the most commonly cited maximum amount (31%), followed by 30 SAR (15%) and 20 SAR (12%) (Table 3). The distribution of WTP values is illustrated in Figure 1.

### Variables Associated with the WTP

Table 4 presents univariable and multivariable logistic regression analyses examining factors associated with willingness to pay (WTP) for community pharmacy-based weight management services. Crude odds ratios (cOR) and adjusted odds ratios (aOR) are reported with 95% confidence intervals. Variables with *p* < 0.20 in univariable analysis were considered for inclusion in the multivariable model.

In the multivariable analysis, monthly income emerged as a significant predictor (*p* = 0.001), with individuals earning 5K–9.9K SAR (aOR = 2.90) and ≥30K SAR (aOR = 2.12) being significantly more likely to pay compared to those earning ≤5K SAR. Access to a registered dietitian was also significant (*p* = 0.006); those who currently pay privately (aOR = 2.71) or desire access (aOR = 2.36) showed higher WTP. The perceived usefulness of pharmacy-based services was the strongest predictor (*p* < 0.001), with those rating services as “useful” having over three times the odds of WTP (aOR = 3.10). In contrast, gender, BMI, marital status, education, and frequency of pharmacy visits were not significant in the adjusted model.

Among participants who reported a positive willingness to pay, linear regression was used to examine factors associated with the maximum amount willing to be paid per session. In the multivariable model, male gender remained independently associated with a lower payment amount, with men willing to pay, on average, 3.8 SAR less per session than women (β = −3.8, 95% CI −6.5 to −1.1; *p* = 0.006). Age group showed borderline evidence of an overall association (*p* = 0.053), with participants aged 45 years or older tending to report lower payment amounts than those aged under 25 years. Frequency of community pharmacy visits also showed a borderline association in the adjusted model (*p* = 0.054), although category-specific estimates were imprecise. BMI was not associated with the amount willing to be paid. Descriptively, the most commonly selected amount was 50 SAR per session (Table 5).

These findings suggest that, among those already willing to pay, women were willing to pay higher amounts than men, whereas differences by BMI and other service-related characteristics were not clearly demonstrated in the adjusted analysis.

Crude ORs (cOR) were estimated from univariable logistic regression models, and adjusted Ors (aOR) were estimated from the multivariable logistic regression model, with willingness to pay (Yes vs. No) as the dependent variable. Reference categories are indicated in the table. Variables with *p* < 0.20 in univariable analysis, together with prespecified covariates, were considered for inclusion in the multivariable model.

Regression coefficients (β) and 95% confidence intervals (CI) were estimated using univariable and multivariable linear regression, with the maximum stated amount willing to be paid per session (SAR) as the dependent variable. Analyses were restricted to participants who reported a positive amount (>0 SAR) (N = 485); observations with missing or zero values were excluded. Reference categories are indicated in the table.

## 4. Discussion

This study evaluated the willingness of a sample of adults attending an academic medical center in Riyadh, Saudi Arabia, to pay for community pharmacist-led weight management services. Among the 746 participants, the majority (66%) expressed willingness to pay (WTP) for such services. The findings indicate that WTP is significantly associated with younger age, single marital status, higher monthly income, and a strong perceived usefulness of pharmacy-based interventions.

Furthermore, engagement with the healthcare system, evidenced by frequent pharmacy visits and prior or desired access to a registered dietitian, was a strong predictor of readiness to pay. Interestingly, clinical burden (such as the presence of chronic diseases or higher BMI) and previous use of weight-loss interventions did not significantly drive WTP. Among those willing to pay, the most frequently selected maximum amount was 50 SAR per session. Additionally, male gender was independently associated with a lower stated payment amount compared to females.

The high rate of WTP observed in this study (66%) aligns with recent findings from the Middle East, though it contrasts with older studies in Western contexts. For instance, a 2024 study by Al-Taani et al. in Jordan (n = 966) reported an even higher WTP rate of 85.2% for similar hypothetical pharmacist-led services. Consistent with our findings, the Jordanian study identified that perceiving the service as useful and having a higher standard of living (analogous to income in our cohort) were significant predictors of WTP [10].

Our findings regarding consumer expectations and barriers also resonate with the international literature. In our cohort, 34% were unwilling to pay, citing a preference for independent weight management and financial concerns as primary reasons. Similarly, Um et al. (2014) [9], in an Australian online survey (n = 403), found that about half of their respondents were unwilling to pay. However, Um et al. noted that consumers who had previously consulted a pharmacist were more willing to pay for future services [9]. While our study found that prior pharmacist consultation for weight/lifestyle advice was not independently associated with WTP in the adjusted model, the frequency of pharmacy visits was a significant driver, underscoring the importance of general pharmacy engagement. In terms of payment amounts, our participants most commonly selected 50 SAR (approximately $13.30 USD) per session. This is comparable to the mean WTP of $17.20 reported in Jordan [10] and the median of AU$10 reported in Australia [9]. This pattern of moderate-to-high consumer WTP for pharmacist-provided services is consistent with the broader global literature. Painter et al. (2018) [18] synthesized 31 contingent valuation studies across diverse settings and found that WTP rates for various pharmacist-provided services ranged from 36% to 95%, with payment amounts spanning from as little as USD $4.02 for services reducing medication-related problems to USD $40 for specialized consultations such as hormone replacement therapy counseling. Notably, their review identified that younger age, higher income, and prior engagement with pharmacist services were the most consistent predictors of WTP across studies, a finding that closely mirrors our own multivariable results, where younger age, higher income, and perceived usefulness were the dominant independent predictors.

This study possesses several notable strengths. It is the first to quantitatively assess WTP for community pharmacist-led weight management services in Saudi Arabia, addressing a critical gap in the literature and providing baseline data for local health policy. The large sample size (n = 746) and the exceptionally high response rate (95.5%) enhance the statistical power and reliability of the findings. A methodological strength of this study is the development of the payment card values. To ensure contextual appropriateness and minimize anchoring or ceiling/floor biases, the payment values were informed by literature and determined through an expert panel. This panel included professionals with deep insights into the Saudi healthcare system—such as experts from NUPCO, the Cooperative Health Insurance (CHI) council, and various insurance companies. Each expert was given the opportunity to propose a value set, and the mean of these proposed values was utilized for the survey. Furthermore, the use of both univariable and multivariable regression models allowed for the careful isolation of independent predictors of WTP.

However, the study is not without limitations. The use of convenience sampling within a single medical city in Riyadh may limit the generalizability of the findings to rural populations or other regions within Saudi Arabia. Furthermore, recruiting participants in a hospital outpatient clinic waiting area (KSUMC) rather than a community pharmacy might introduce selection bias, as hospital attendees may have a different health consciousness or disease burden compared to the general pharmacy-visiting public, and may therefore exhibit different WTP patterns.

Our cohort was predominantly young (45% under 25 years), female (64%), and highly educated (74% with a university degree). This demographic profile may introduce an upward bias in the reported WTP estimates, as higher education levels are consistently associated with greater health service valuation. Additionally, the WTP assessment was based on a hypothetical scenario; stated WTP does not always translate to actual purchasing behavior in real-world settings. Finally, anthropometric data (BMI) and chronic conditions were self-reported, which introduces the potential for recall or social desirability bias. Future studies should employ stratified sampling across multiple geographic regions and socioeconomic groups to produce nationally representative WTP estimates.

From a patient perspective, the moderate price point of 50 SAR per session suggests that pharmacist-led weight management services are financially accessible to a substantial proportion of the population; however, the strong association between income and WTP (aOR 2.90) highlights that lower-income patients may face financial barriers, warranting subsidized or tiered pricing models. For healthcare providers, the finding that perceived usefulness, rather than clinical need such as BMI or chronic disease, is the primary driver of WTP underscores the need for pharmacists to actively communicate and demonstrate the value of their services to patients, supported by evidence that pharmacist-led weight management interventions are effective in reducing body weight across diverse settings [15]. From a policy perspective, these findings provide the first consumer-validated pricing benchmark for pharmacist-led weight management in Saudi Arabia, offering actionable data to support the formalization and reimbursement of such services within the Vision 2030 healthcare transformation agenda, particularly as obesity has escalated to the second leading risk factor for disease burden in the Kingdom.

## 5. Conclusions

Among adults attending a large academic medical city in Riyadh, there is a substantial willingness to pay for community pharmacist-led weight management services, particularly among younger, higher-income individuals who actively engage with pharmacies and perceive these services as useful. Financial commitment is moderate but clear, with 50 SAR per session being the most acceptable price point. The findings suggest that clinical need alone (e.g., BMI or chronic disease) is insufficient to drive investment in these services; rather, perceived value and existing healthcare engagement are the primary catalysts. To successfully integrate weight management programs into Saudi community pharmacies, stakeholders may consider focusing on demonstrating the tangible value of these services, ensuring affordability, and leveraging the existing trust between patients and pharmacists. Future initiatives should consider these consumer preferences to design culturally tailored, patient-centered, and economically viable obesity interventions.

## Figures and Tables

**Table 1:** Demographic characteristics.

Characteristic	N = 746
Gender	
Female	479 (64%)
Male	267 (36%)
Age	
Mean (SD)	31.7 (12.4)
Age groups	
<25	333 (45%)
25–34	150 (20%)
35–44	116 (16%)
45+	147 (20%)
BMI (kg/m^2^)	
Mean (SD)	25.3 (5.6)
BMI Category	
Underweight (<20)	117 (16%)
Normal weight (20–24)	289 (39%)
Overweight (25–29)	213 (29%)
Obese (30–39)	116 (16%)
Morbidly obese (>40)	11 (1.5%)
Any comorbidity	
No	557 (75%)
Yes	184 (25%)
Weight-management medication use	
No	712 (95%)
Yes	34 (4.6%)
Marital status	
Single	428 (57%)
Married	299 (40%)
Divorced	14 (1.9%)
Widow	5 (0.7%)
Occupation	
Medical field	80 (11%)
Non-medical field	302 (40%)
Not employed	364 (49%)
Education level	
No formal education	3 (0.4%)
Primary	7 (0.9%)
Middle school	11 (1.5%)
Secondary	117 (16%)
Higher education	53 (7.1%)
University degree	555 (74%)
Health insurance	
No	476 (64%)
Yes	270 (36%)
Monthly income (in SAR)	
≤5K	309 (41%)
5K–9.9K	133 (18%)
10K–14.9K	132 (18%)
15K–19.9K	76 (10%)
20K–24.9K	44 (5.9%)
25K–29.9K	21 (2.8%)
≥30K	31 (4.2%)
Living area	
Rural	36 (4.8%)
Urban	710 (95%)

**Table 2:** Current and Preferred Weight Management Support Services.

Characteristic	Overall N = 746 ^1^	Willingness to Pay		*p*-Value ^2^
		Yes N = 495 ^1^	No N = 251 ^1^	
What type of weight management support do you currently receive?				<0.001
None	472 (51%)	300 (48%)	172 (59%)	
Behavioral counseling	29 (3.2%)	23 (3.7%)	6 (2.1%)	
Online programs/apps (MyFitnessPal, Pacer)	141 (15%)	117 (19%)	24 (8.3%)	
Pharmacist consultations	35 (3.8%)	25 (4.0%)	10 (3.4%)	
Physician consultations	101 (11%)	69 (11%)	32 (11%)	
Registered dietitian sessions	99 (11%)	63 (10%)	36 (12%)	
Support groups (WhatsApp or Telegram groups)	40 (4.4%)	30 (4.8%)	10 (3.4%)	
What format would you prefer for weight management consultations?				<0.001
Individual one-on-one consultations	431 (49%)	313 (53%)	118 (41%)	
Virtual/telehealth consultations	281 (32%)	201 (34%)	80 (28%)	
Not applicable	162 (19%)	75 (13%)	87 (31%)	
What services would you expect to be included in a weight management consultation?				>0.9
Advice on nutrition	371 (16%)	276 (16%)	95 (16%)	
Behavioral support	215 (9.2%)	160 (9.2%)	55 (9.4%)	
Goal setting and progress tracking	239 (10%)	179 (10%)	60 (10%)	
Lifestyle recommendations (physical activity, healthy eating habits…etc.)	742 (32%)	556 (32%)	186 (32%)	
Medication counseling and side effect management	326 (14%)	247 (14%)	79 (13%)	
Body weight and BMI monitoring	438 (19%)	327 (19%)	111 (19%)	

^1^ n (%). ^2^ Pearson’s Chi-squared test; Fisher’s exact test.

**Table 3:** Willingness to Pay for Pharmacy-Based Weight Management Services.

Characteristic	Overall N = 746 ^1^	*p*-Value ^2^
Would you be willing to pay for this program?		<0.001
No	251 (34%)	
Yes	495 (66%)	
If no, why not?		0.11
Already managing weight independently	98 (39%)	
Doubt pharmacists’ qualifications or motives	4 (1.6%)	
Financial or insurance concerns	52 (21%)	
Not applicable	36 (14%)	
Not interested	53 (21%)	
Prefer nutrition specialist	2 (0.8%)	
Prefer physician or doctor	3 (1.2%)	
Use social media instead	3 (1.2%)	
What is the maximum amount you would pay per session?		0.5
0	11 (2.2%)	
15	36 (7.3%)	
20	60 (12%)	
30	75 (15%)	
35	59 (12%)	
40	52 (10%)	
50	152 (31%)	
55	11 (2.2%)	
65	40 (8.1%)	

^1^ n (%). ^2^ Pearson’s Chi-squared test; Fisher’s exact test.

**Table 4:** Univariable and multivariable logistic regression analyses of factors associated with willingness to pay for community pharmacy-based weight management services.

Variable	Univariable			Multivariable		
	cOR	95% CI	*p*	aOR	95% CI	*p*
Gender			0.2			0.056
Female	Ref	Ref		Ref	Ref	
Male	0.80	0.58, 1.10		0.70	0.49, 1.01	
Age group			<0.001			0.078
<25	Ref	Ref		Ref	Ref	
25–34	0.62	0.41, 0.94		0.50	0.29, 0.88	
35–44	0.61	0.39, 0.97		0.55	0.24, 1.21	
45+	0.40	0.26, 0.59		0.43	0.19, 0.95	
BMI	0.96	0.94, 0.99	0.004	0.99	0.96, 1.02	0.4
Marital status			0.003			>0.9
Single	Ref	Ref		Ref	Ref	
Married	0.55	0.40, 0.76		0.90	0.48, 1.68	
Divorced	0.71	0.24, 2.36		1.29	0.34, 5.40	
Widow	0.59	0.10, 4.54		1.01	0.12, 9.99	
Occupation			0.2			
Medical field	Ref	Ref				
Non-medical field	1.09	0.65, 1.81				
Not employed	1.44	0.87, 2.37				
Education level			0.9			
No formal education	Ref	Ref				
Primary	0.67	0.02, 10.9				
Middle school	0.60	0.02, 8.25				
Secondary	0.84	0.04, 8.99				
Higher education	0.97	0.04, 10.8				
University degree	1.02	0.05, 10.8				
Health insurance			0.4			
No	Ref	Ref				
Yes	1.14	0.83, 1.58				
Monthly income SAR			0.001			0.001
≤5K	Ref	Ref	Ref	Ref	Ref	
5K–9.9K	1.88	0.99, 3.87		2.90	1.39, 6.49	
10K–14.9K	1.17	0.68, 2.04		0.83	0.46, 1.54	
15K–19.9K	0.41	0.20, 0.80		0.47	0.21, 0.95	
20K–24.9K	0.61	0.25, 1.24		0.66	0.26, 1.42	
25K–29.9K	0.63	0.29, 1.24		0.76	0.34, 1.59	
≥30K	1.75	1.01, 3.07		2.12	1.16, 3.89	
Chronic conditions			0.5			
No	Ref	Ref				
Yes	0.89	0.63, 1.26				
Weight medication use			0.9			
No	Ref	Ref				
Yes	0.94	0.46, 1.98				
Any procedures to lose weight			0.7			
No	Ref	Ref				
Yes	0.91	0.59, 1.43				
Do you currently have access to a registered dietitian?			<0.001			0.006
No, and I’m not interested	Ref	Ref		Ref	Ref	
No, but I would like access	2.30	1.44, 3.71		2.36	1.40, 4.01	
Not sure	1.31	0.75, 2.28		1.47	0.80, 2.71	
Yes, I pay privately	2.94	1.67, 5.28		2.71	1.45, 5.16	
Yes, through my healthcare provider	1.69	1.08, 2.62		1.69	1.04, 2.76	
How often do you visit a community pharmacy?			<0.001			0.002
Daily	Ref	Ref		Ref	Ref	
Weekly	3.21	0.70, 14.8		2.34	0.44, 12.5	
Monthly	2.83	0.65, 12.3		2.12	0.42, 10.6	
Every few months	1.56	0.36, 6.81		1.08	0.21, 5.44	
Rarely	1.42	0.33, 6.15		1.02	0.20, 5.11	
Have you ever asked a pharmacist for help with weight, diet or lifestyle?			0.12			0.6
No	Ref	Ref		Ref	Ref	
Yes	1.42	0.91, 2.25		1.16	0.71, 1.95	
How useful do you think pharmacy-based weight management services are?			<0.001			<0.001
Not useful	Ref	Ref		Ref	Ref	
Neutral	1.55	0.98, 2.43		1.43	0.87, 2.35	
Useful	3.72	2.29, 6.05		3.10	1.83, 5.26	

**Table 5:** Univariable and multivariable linear regression analyses of factors associated with the maximum amount willing to be paid per session for community pharmacy-based weight management services among participants willing to pay (N = 485).

Variable	Univariable			Multivariable		
	Beta	95% CI	*p*	Beta	95% CI	*p*
Gender			0.005			0.006
Female	Ref	Ref		Ref	Ref	
Male	−3.8	−6.5, −1.2		−3.8	−6.5, −1.1	
Age group			0.023			0.053
<25	Ref	Ref		Ref	Ref	
25–34	0.05	−3.3, 3.4		0.91	−2.5, 4.3	
35–44	2.3	−1.4, 6.0		2.7	−1.2, 6.6	
45+	−4.5	−8.2, −0.92		−3.5	−7.4, 0.30	
BMI	−0.13	−0.36, 0.10	0.3	−0.06	−0.31, 0.19	0.6
Marital status			0.4			
Single	Ref	Ref				
Married	−0.74	−3.4, 1.9				
Divorced	−8.4	−18, 1.1				
Widow	−2.8	−19, 13				
Monthly income SAR			0.2			
≤5K						
5K–9.9K	5.9	1.2, 11				
10K–14.9K	0.93	−3.4, 5.3				
15K–19.9K	−0.52	−5.1, 4.1				
20K–24.9K	0.64	−4.3, 5.6				
25K–29.9K	−0.36	−4.8, 4.1				
≥30K	−2.4	−6.7, 1.8				
Do you currently have access to a registered dietitian?			0.13			0.10
No, and I’m not interested	Ref	Ref		Ref	Ref	
No, but I would like access	0.55	−3.6, 4.7		0.17	−4.0, 4.3	
Not sure	−0.55	−5.7, 4.6		−0.72	−5.8, 4.4	
Yes, I pay privately	4.4	−0.27, 9.0		4.5	−0.15, 9.1	
Yes, through my healthcare provider	−0.58	−4.6, 3.5		−0.61	−4.7, 3.5	
How often do you visit a community pharmacy?			0.009			0.054
Daily	Ref	Ref		Ref	Ref	
Weekly	3.3	−9.6, 16		3.6	−9.2, 16	
Monthly	−3.7	−16, 8.8		−2.4	−15, 10	
Every few months	−0.25	−13, 12		0.26	−12, 13	
Rarely	0.33	−12, 13		0.71	−12, 13	
Have you ever asked a pharmacist for help with weight, diet or lifestyle?			>0.9			
No	Ref	Ref				
Yes	0.16	−3.3, 3.6				
How useful do you think pharmacy-based weight management services are?			0.6			
Not useful	Ref	Ref				
Neutral	−2.4	−6.8, 2.0				
Useful	−2.2	−6.5, 2.2				

## Data Availability

The data presented in this study are available on request from the corresponding author due to ethical and confidentiality considerations related to participant survey data.

## Supplementary Materials

The following supporting information can be downloaded at: https://www.mdpi.com/article/10.3390/healthcare14131953/s1, File S1. Topic: Willingness to Pay for a Weight Management services in a community pharmacy. File S2. Prevalence and Types of Self-Reported Chronic Health Conditions Among Participants. File S3. Community Pharmacy Utilization and Perceptions of Pharmacy-Based Weight Management Services. File S4. Use of Prescription Weight Management Medications and Weight Loss Procedures.
