# Supplementary material for: Willingness to Pay for Pharmacist-Led Weight Management Services in Community Pharmacies: A Cross-Sectional Study in an Academic Medical Center in Saudi Arabia
Source: Healthcare (Basel). 2026 Jul 2;14(13):1953. doi: 10.3390/healthcare14131953 (PMC13360955; doi:10.3390/healthcare14131953)
Supplement: Supplementary file 1 [file healthcare-14-01953-s001.zip › healthcare-4357941-supplementary.pdf]

Supplementary File S1

Topic: Willingness to Pay for a Weight Management services in a community pharmacy

Section 1: Demographic Information (1)

1. **Gender:** ☐ Male ☐ Female (1)
2. **Age:** _____ years (1)
3. **Marital Status:** ☐ Single ☐ Married (1)
4. **Occupation:** ☐ Not employed ☐ Medical field ☐ Non- medical field
5. **Education level:**

   ☐ No formal education ☐ Primary ☐ Secondary ☐ University degree ☐ Higher education (1)

6. **Health Insurance:** ☐ Yes ☐ No (1)
7. **Monthly Income (SAR):**

   ☐ ≤5K

   ☐ 5K – 9.9K

   ☐ 10K – 14.9K

   ☐ 15K – 19.9K

   ☐ 20K - 24.9K

   ☐ 25K - 29.9K

   ☐ ≥30K

8. **Living area:** ☐ Urban ☐ Rural (1)
9. **Self-reported weight:** _______ kg (1)

10. **Self-reported height: _______** cm (1)

11. Any chronic condition(s)?

☐ Diabetes

☐ Hypertension

☐ CVD

☐ asthma

☐ cancer

☐ Other: _______

☐ None

Section 2: Access to obesity medications:

12. Do you currently have access to a registered dietitian?

☐ Yes, through my healthcare provider

☐ Yes, I pay privately

☐ No, but I would like access

☐ No, and I'm not interested

☐ Not sure

13. Are you currently using any prescription medications for weight management/obesity?

☐ Yes ☐ No

14. If yes, which type of medication are you using? (Check all that apply)

☐ GLP-1 receptor agonists (e.g., semaglutide: Wegovy or Ozempic or rybelsus, liraglutide: Saxenda)

☐ Dual GIP/GLP-1 receptor agonist (tirzepatide: Zepbound or Mounjaro)

☐ Lipase inhibitor (orlistat: Alli, Xenical)

☐ Naltrexone–bupropion ER (Contrave)

☐ Other (please specify): ___________

15. How long have you been using obesity medication?

☐ Less than 3 months

☐3- less than 6 months

☐6- less than12 months

☐1-less than 2 years

☐ More than 2 years

☐ Not applicable

16. Which of the following weight loss procedures have you undergone?

☐ Gastric sleeve

☐ Gastric bybass

☐ Other (please specify): ___________

☐ None

Section 3: Experience and Perception (1,2)

17. **How often do you visit a community pharmacy?**

    ☐ Daily ☐ Weekly ☐ Monthly ☐ every few months ☐ Rarely

18. **Have you ever asked a pharmacist for help with weight, diet, or lifestyle? (2)**

    ☐ Yes ☐ No

If yes:

19. **What kind of help did you receive? (2)**

    ☐ Advice on nutrition

    ☐ Product recommendation

    ☐ Lifestyle tips

    ☐ Monitoring progress

20. **How useful do you think pharmacy-based weight management services are? (2)**

    ☐ Very useful ☐ Useful ☐ Neutral ☐ Not useful ☐ Very not useful

21. **What type of weight management support do you currently receive? (Check all that apply)**

    ☐ Physician consultations

    ☐ Registered dietitian sessions

    ☐ Behavioral counseling

☐ Pharmacy consultations (side effects, dosage recommendations)

☐ Online programs/apps (Myfitness pal, pacer)

☐ Support groups (Whatsapp or telegram groups)

☐ None

Section 4: Expectations and preference

22. **What format would you prefer for weight management consultations? (Check all that apply)**

☐ Individual one-on-one consultations ☐ Virtual/telehealth consultations

23. **What services would you expect to be included in a weight management consultation? (Check all that apply)**

☐ Weight and BMI monitoring

☐ Medication counseling and side effect management

☐ Nutritional guidance

☐ lifestyle recommendations (physical activity, healthy eating habits...etc)

☐ Behavioral support

☐ Goal setting and progress tracking

Willingness to Pay (WTP) hypothetical scenario (1,2)

Imagine a weight management service provided in your nearest community pharmacy to help you achieve and maintain optimal weight and improve your health.

Evidence showed that people who received pharmacist-led weight management support lost an average of nearly 5 kg, reduced their BMI by about 1.3 kg/m², and decreased their waist circumference by almost 5 cm within six months.

The proposed service in Saudi community pharmacies would include:

- Consultation (30–45 minutes) with a trained pharmacist.
- Medication review and optimization.
- Recommendation of over-the-counter supplements if needed.
- Health and lifestyle assessment.
- InBody analysis (weight, height, BMI, body fat percentage).
- Nutrition and exercise counselling.
- Educational materials in Arabic.
- Goal setting and progress tracking.
- <u>Service cost does not cover any medications.</u>

24. Would you be willing to pay for this program?

☐ Yes ☐ No

If no, why not?

☐ Financial constraints

☐ Already managing weight on my own

☐ Not interested

☐ Other

25. What is the maximum amount you would pay per session? (1,2)

0	15	20
30	35	40
50	55	65

Supplementary File S2

Prevalence and Types of Self-Reported Chronic Health Conditions Among Participants

**Characteristic**	**Overall** N = 746[1]	**Willing to pay**		**p-value**[2]
		**Yes** N = 495[1]	**No** N = 251[1]	
**Have chronic conditions**				0.6
No	557 (75%)	372 (76%)	185 (74%)	
Yes	184 (25%)	118 (24%)	66 (26%)	
Adrenal Gland Disorder	1	1	0	
Anemia	3	1	2	
Asthma	46	35	11	
Blood Diseases	1	1	0	
Cancer	2	1	1	
Cholesterol	4	0	4	
Crohn's Disease	4	1	3	
CVD	14	8	6	
Depression	1	0	1	
Diabetes	49	30	19	
Epilepsy	1	1	0	
Fatty Liver	1	1	0	
GAD	2	1	1	

Hypertension	47	25	22	
Immune Diseases	2	2	0	
Kidney Failure	1	1	0	
Migraine	2	1	1	
Multiple Sclerosis	2	2	0	
PCOS	1	1	0	
Prostate	1	1	0	
Rheumatoid Arthritis	3	1	2	
Stomach Disorders	1	1	0	
Thyroid disorders	30	18	12	
Urticaria / Fibromyalgia	1	1	0	

[1] n (%)

[2] Pearson's Chi-squared test.

CVD: Cardiovascular Diseases; GAD: Generalized Anxiety Disorder; PCOS: Polycystic Ovary Syndrome.

Supplementary File S3

Community Pharmacy Utilization and Perceptions of Pharmacy-Based Weight Management Services

**Characteristic**	**Overall N = 746**[1]	**Willing to pay**		**p-value**[2]
		**Yes N = 495**[1]	**No N = 251**[1]	
**Frequency of community pharmacy visits**				<0.001
Daily	9 (1.2%)	5 (1.0%)	4 (1.6%)	
Weekly	80 (11%)	61 (12%)	19 (7.6%)	
Monthly	251 (34%)	186 (38%)	65 (26%)	
Every few months	169 (23%)	103 (21%)	66 (26%)	
Rarely	237 (32%)	140 (28%)	97 (39%)	
**Asked pharmacist for weight/diet/lifestyle help**				0.11
No	635 (85%)	414 (84%)	221 (88%)	
Yes	111 (15%)	81 (16%)	30 (12%)	
**If yes what kind of help did you receive**				0.5
Advice on nutrition	68 (34%)	48 (32%)	20 (41%)	
Lifestyle tips	43 (22%)	32 (21%)	11 (22%)	

Monitoring progress	22 (11%)	16 (11%)	6 (12%)	
Product recommendation	65 (33%)	53 (36%)	12 (24%)	
**Perceived usefulness of pharmacy-based services**				<0.001
Not useful	100 (13%)	51 (10%)	49 (20%)	
Neutral	347 (47%)	209 (42%)	138 (55%)	
Useful	299 (40%)	235 (47%)	64 (25%)	
**Access to a registered dietitian**				<0.001
No, and I'm not interested	126 (17%)	67 (14%)	59 (24%)	
No, but I would like access	190 (25%)	138 (28%)	52 (21%)	
Not sure	88 (12%)	53 (11%)	35 (14%)	
Yes, I pay privately	104 (14%)	80 (16%)	24 (9.6%)	
Yes, through my healthcare provider	238 (32%)	157 (32%)	81 (32%)	

[1] n (%)

[2] Pearson's Chi-squared test; Fisher's exact test.

Supplementary File S4

Use of Prescription Weight Management Medications and Weight Loss Procedures

Characteristic	Overall N = 746[1]	Willing to pay		p-value[2]
		Yes N = 495[1]	No N = 251[1]	
**Currently using prescription medication**				>0.9
No	712 (95%)	473 (96%)	239 (95%)	
Yes	34 (4.6%)	22 (4.4%)	12 (4.8%)	
**Specify weight medication**				0.066
Dual GIP/GLP-1 receptor agonist (tirzepatide)	14 (40%)	12 (55%)	2 (15%)	
GLP-1 receptor agonists	13 (37%)	7 (32%)	6 (46%)	
Metformin	3 (8.6%)	1 (4.5%)	2 (15%)	
Naltrexone–bupropion ER	1 (2.9%)	1 (4.5%)	0 (0%)	
Orlistat	4 (11%)	1 (4.5%)	3 (23%)	
**Duration of obesity medication use**				0.5
Less than 3 months	15 (2.0%)	9 (1.8%)	6 (2.4%)	
3 - 6 months	7 (0.9%)	6 (1.2%)	1 (0.4%)	
6 - 12 months	6 (0.8%)	5 (1.0%)	1 (0.4%)	
1- 2 years	7 (0.9%)	3 (0.6%)	4 (1.6%)	
More than 2 years	1 (0.1%)	1 (0.2%)	0 (0%)	

**Any procedures to lose weight**				0.6
No	648 (87%)	432 (87%)	216 (86%)	
Yes	98 (13%)	63 (13%)	35 (14%)	
**Type of procedure to lose weight**				0.2
Bariatric Surgery	35 (31%)	28 (38%)	7 (18%)	
Diet	43 (38%)	24 (32%)	19 (48%)	
Exercise	20 (18%)	12 (16%)	8 (20%)	
Fasting	11 (9.6%)	7 (9.5%)	4 (10%)	
Injectable weight loss medications	5 (4.4%)	3 (4.1%)	2 (5.0%)	

[1] n (%)

[2] Pearson's Chi-squared test; Fisher's exact test.

References:

1. Al-Taani G, Arabyat R, Mousa R. Perceptions about a pharmacy-delivered weight management service in community pharmacy settings in Jordan. J Appl Pharm Sci. 2024 Mar 1;14(3):191–8.
2. Um IS, Armour C, Krass I, Gill T, Chaar BB. Consumer perspectives about weight management services in a community pharmacy setting in NSW, Australia. Health Expectations. 2014;17(4):579–92.
